# Hierarchical multi-agent reinforcement learning for retrieval-augmented industrial document question answering

**DOI:** 10.1038/s41598-026-41684-z

**Published:** 2026-03-14

**Authors:** Yihong Qian, Baoli Han, Yufeng Yuan, Xiaofeng Zhang, Hang Zhu, Li Ni, Shaojun Zhong

**Affiliations:** 1 State Grid Zhejiang Electric Power Co., Ltd., Shaoxing Power Supply Company, Shaoxing, Zhejiang China; 2 State Grid Zhejiang Electric Power Co., Ltd., Shengzhou Power Supply Company, Shengzhou, Zhejiang China

**Keywords:** Multimodal documents, Retrieval-augmented generation, Multi-agent reinforcement learning, Adaptive retrieval, Industrial document understanding, Engineering, Mathematics and computing

## Abstract

Multimodal industrial documents–such as operation manuals, circuit diagrams, and parameter tables–contain domain knowledge distributed across text, images, and document layout. However, most existing retrieval-augmented generation (RAG) frameworks rely on static retrieval and fusion policies with fixed modality weights and uniform retrieval depth, making them less adaptable to diverse query intents and dynamic cross-modal dependencies. As a result, they often retrieve incomplete evidence and yield suboptimal reasoning in complex long-document scenarios. To address these challenges, we propose MARL-RAGDoc, a hierarchical multi-agent reinforcement learning framework for multimodal retrieval-augmented reasoning. A high-level coordinator agent dynamically allocates modality weights and retrieval depth based on query characteristics, while specialized text, image, and table agents perform fine-grained evidence selection within their respective candidate pools. A collaborative reasoning module integrates the retrieved evidence and provides hierarchical reward signals to continuously optimize retrieval policies. Experimental results on multiple multimodal document benchmarks demonstrate that MARL-RAGDoc consistently outperforms baselines in both retrieval accuracy and reasoning performance, while remaining computationally efficient. Our code and dataset are publicly available at https://github.com/Yihong-Q/MARL-RAGDoc.

## Introduction

In typical industrial scenarios such as power systems, a large collection of multimodal documents, including operation manuals, maintenance logs, circuit diagrams, and parameter tables, encodes domain knowledge^1–3^. These documents often contain text, images, tables, and structured symbols, with complex semantics and strong domain constraints^4,5^. Effectively using these resources remains challenging for equipment operation, dispatch decision-making, and safety management^6^. However, current approaches to knowledge extraction and reasoning over industrial documents still rely heavily on manual effort, which is time-consuming and costly. To address this, researchers have begun exploring retrieval-augmented techniques that integrate retrieval and generation, aiming to achieve automated reasoning while maintaining factual accuracy and contextual relevance^7^.

Retrieval-Augmented Generation (RAG) was initially proposed as a general framework that combines neural retrieval with generative models to enhance factuality and contextual coherence in text generation^8^. With the recent progress of large language models, RAG has incorporated adaptive retrieval, tool-augmented reasoning, and reflection-based generation strategies, which help reduce hallucinations and improve grounding and interpretability^9,10^. Although these methods improve factuality and explainability in text generation tasks, they remain primarily focused on single-modal text and are insufficient for handling multimodal industrial documents containing images, tables, and complex layouts. Therefore, researchers have begun exploring the extension of RAG to multimodal document understanding to better support cross-modal reasoning for complex tasks.

Representative work such as LayoutLM and DocFormer^2,11^ leverages layout-aware visual-language pretraining to jointly model textual, visual, and layout features, enabling multimodal semantic understanding. Some studies further combine retrieval and generation for tasks such as visual-question answering or multimodal summarization to enhance factual consistency and contextual comprehension^12,13^. In complex scenarios such as the power industry, where document types and task granularities vary greatly, static fusion strategies fail to simultaneously balance semantic relevance, context completeness, and reasoning stability, limiting model generalization and interpretability in real-world industrial environments.

**Fig. 1:**
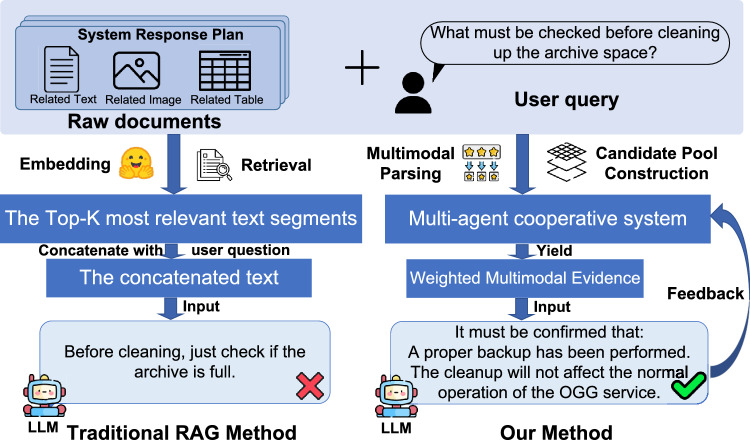
Comparison of the conventional RAG pipeline and our hierarchical multi-agent multimodal framework (MARL-RAGDoc).

Overall, current research on multimodal RAG faces two key challenges: (1) Dynamic modality dependence. Different tasks rely on different modalities for example, images dominate spatial reasoning while tables govern parameter computation and trend analysis; fixed modality weights and Top-*K* retrieval cannot adapt to these shifts. (2) Disconnected retrieval and reasoning. Existing systems typically optimize retrieval and generation independently, preventing reasoning feedback from guiding retrieval strategies, which leads to insufficient coverage and redundant information. Figure 1 contrasts the traditional RAG pipeline with our multi-agent multimodal framework, highlighting the need for adaptive modality coordination and feedback-driven retrieval.

To address these challenges, we introduce a Hierarchical Multi-Agent Reinforcement Learning (MARL) framework for multimodal retrieval-augmented reasoning, termed **MARL-RAGDoc**. MARL-RAGDoc follows a four-stage pipeline: (1) *Multimodal parsing* decomposes documents into layout-aware text blocks, images, and tables with positional metadata; (2) *Candidate pool construction* embeds these elements into a unified space and builds modality-specific Top-*K* candidate pools for the query; (3) *Hierarchical MARL retrieval* uses a coordinator agent to dynamically set modality weights and retrieval depth, while modality-specific agents iteratively select and expand evidence within each pool; (4) *Collaborative reasoning and reflection* generates an answer from weighted multimodal evidence and uses answer quality as hierarchical reward signals to refine retrieval policies in a closed retrieve–reason–reflect loop. This staged design is particularly useful for industrial multimodal documents, where queries require flexible coordination across modalities.

The main contributions of this work are as follows:

- **MARL-RAGDoc** is introduced as a hierarchical multi-agent architecture that unifies multimodal retrieval, reasoning, and reflection into a closed-loop framework.
- The framework includes task-conditioned modality weighting, adaptive retrieval-depth control, retrieval–reasoning co-optimization, and layout-aware multimodal alignment for cross-modal inference.
- Extensive experiments on real-world multimodal industrial datasets demonstrate that MARL-RAGDoc improves retrieval efficiency, reasoning accuracy, and interpretability over strong baselines.

## Related work

### Retrieval-augmented generation

Retrieval-Augmented Generation (RAG) has emerged as a central paradigm for improving factual grounding and reducing hallucinations in large language models by incorporating external evidence during generation. The seminal architecture proposed by Lewis et al.^14^ retrieves semantically relevant passages from a knowledge corpus and conditions the decoder on both the query and retrieved documents, thereby improving performance on knowledge-intensive tasks such as open-domain question answering.

Subsequent research has explored more adaptive and self-regulating retrieval mechanisms. Self-RAG^15^ equips language models with the ability to autonomously decide when to retrieve and critique their own outputs via reflection tokens, leading to more reliable factual grounding. Building on this direction, CRAG^16^ introduces a lightweight document evaluator that detects insufficient retrieval and triggers additional web search when necessary. In high-stakes domains like medicine, Wang et al.^17^ proposed LINS, a framework for improving response credibility by continuously gathering up-to-date knowledge and generating evidence-traceable outputs. Jiang et al.^18^ reduced computational redundancy by proposing Active RAG, which learns selective retrieval behaviors conditioned on query complexity. Similarly, Wang et al.^19^ proposed a dual-channel retrieval mechanism that integrates structured knowledge graphs into the RAG architecture to enhance semantic reasoning and mitigate factual hallucinations.

Another line of work combines retrieval with tool use. Toolformer^9^ trains models to invoke external tools through self-supervision, while ReAct^20^ couples chain-of-thought reasoning with actionable tool calls, offering better interpretability and problem-solving capability. Despite these advances, most existing RAG frameworks remain primarily text-centric and employ fixed retrieval policies that do not adapt to multimodal information distributions. This limits their capacity to process complex industrial documents where relevant content may be distributed across diverse modalities such as text regions, tables, diagrams, and layout structures.

### Multimodal document understanding

Multimodal document understanding seeks to jointly model textual content, visual appearance, and document layout to achieve holistic semantic comprehension. The LayoutLM series^1,11,21^ pioneered the integration of text, layout coordinates, and image embeddings through joint pretraining, thereby advancing tasks such as form understanding and document visual question answering. Models like DocFormer^2^ and UDOP^22^ further enhanced spatial-semantic encoding through multimodal self-attention and unified encoder-decoder architectures. Instruction-tuned multimodal LLMs, such as mPLUG-DocOwl^23^ and Kosmos-2.5^24^, have demonstrated strong generalization across document-centric tasks by introducing unified representation spaces for both text-intensive and vision-intensive documents.

Integrating retrieval with multimodal reasoning has become increasingly important. Hu et al.^25^ proposed mRAG to jointly optimize cross-modal retrieval and generation, while VisRAG^26^ employs vision-language models as multimodal retrievers for image-encoded documents. Beyond visual modalities, Fang et al.^27^ extended RAG to the audio domain by combining speech-to-text tools with local LLMs to evaluate simulated teaching voice data, demonstrating the versatility of retrieval-augmented frameworks in handling diverse modalities. In domain-specific applications such as power grids, specialized challenges arise from heterogeneous technical documentation containing single-line diagrams, relay protection schematics, and tabular operational data^28,29^. Recent work has explored multimodal approaches for power system documents: Yang et al.^30^ introduced vision-language models for interpreting electrical diagrams, and Wang et al.^31^ developed document question-answering systems combining OCR with layout-aware transformers.

Nevertheless, existing multimodal RAG systems generally rely on static fusion mechanisms such as fixed modality weights or non-adaptive attention, making them insufficient for complex documents where modality relevance varies dynamically with query intent. Furthermore, domain-specific document systems typically operate in isolation without leveraging cross-document reasoning or hierarchical information aggregation—a major limitation for diagnostic tasks requiring synthesis across multiple technical manuals and operational records.

### Multi-agent reinforcement learning

Multi-agent reinforcement learning (MARL) provides a principled paradigm for coordinating distributed decision-making, especially in environments requiring both global planning and local specialization. Hierarchical MARL approaches, such as Feudal Networks^32^, introduce hierarchical goal-setting where a manager agent issues abstract goals to worker agents, enabling effective temporal abstraction and credit assignment. Value-decomposition frameworks, including QMIX^33^ and QTRAN^34^, allow decentralized execution while maintaining centralized training signals to ensure cooperative behavior between agents.

Recent work has begun to explore MARL in information-seeking and language-based scenarios. Zhang et al.^35^ proposed a multi-agent conversational information-seeking system where agents specialize in document retrieval, reasoning, and synthesis. AgentCF^36^ applies collaborative MARL to conversational recommendation, demonstrating improved modeling of user preferences. Reinforcement learning has also been used to optimize retrieval strategies in RAG pipelines. Adaptive-RAG^37^ learns retrieval strategies tailored to query difficulty, while Fang et al.^38^ introduced REANO with multi-step retrieval-enhanced reasoning to improve answer accuracy.

However, these methods remain limited to unimodal text-based retrieval and do not address the challenge of coordinating retrieval across heterogeneous modalities. To the best of our knowledge, no prior work systematically studies hierarchical MARL for adaptive multimodal retrieval-augmented generation. Our proposed MARL-RAGDoc framework fills this gap by introducing a hierarchical agent architecture in which a coordinator agent adaptively allocates retrieval depth and modality relevance, while modality-specific agents perform targeted retrieval and fusion. This yields a closed-loop system that jointly optimizes multimodal retrieval and reasoning through hierarchical reward propagation.

## Methodology

**Fig. 2:**
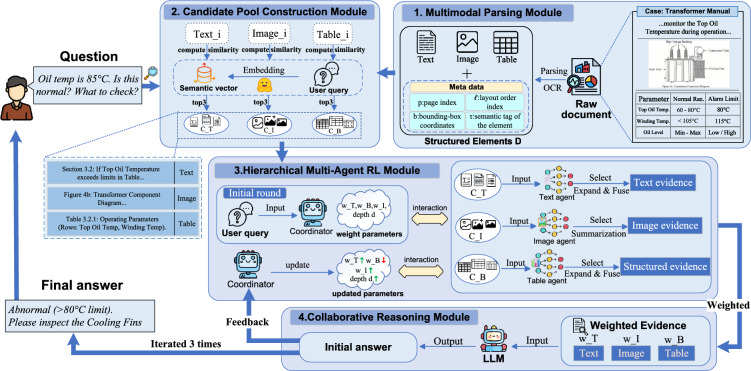
Overview of the proposed MARL-RAGDoc framework, consisting of multimodal parsing, candidate pool construction, hierarchical multi-agent RL retrieval, and collaborative reasoning with a feedback loop.

### Overall framework

Industrial document QA requires adaptive retrieval across text, images, and tables to handle complex layouts. To this end, MARL-RAGDoc follows a “decompose–retrieve–reason” workflow and dynamically orchestrates multiple agents for evidence selection and synthesis. As shown in Fig. 2, the framework comprises four modules:

1. The Multimodal Parsing Module converts raw documents into layout-aware text, image, and table elements. It preserves positional metadata to retain the spatial context required for downstream reasoning.
2. The Candidate Pool Construction Module maps heterogeneous elements into a unified latent space and forms modality-specific Top-*K* candidate pools for the query, providing high-recall retrieval candidates.
3. The Hierarchical Multi-Agent RL Module is the decision-making core. A high-level coordinator adapts the global retrieval strategy (e.g., modality weighting and retrieval depth), while modality-specific agents perform fine-grained selection and intra-modal expansion via lightweight communication.
4. The Collaborative Reasoning Module synthesizes prioritized evidence into a weighted context for answer generation. It further supports an iterative reflection loop that triggers additional retrieval when evidence is insufficient, enabling closed-loop policy optimization.

### Problem formulation

Formally, a document is represented as a set of structured elements:

1 $$\begin{aligned} \mathscr {E} = \{ e_1, e_2, \dots , e_N \}. \end{aligned}$$

where each element $e_i$ is associated with a modality type $\mathbb {T}(e_i) \in \{\text {Text}, \text {Image}, \text {Table}\}$. Given a query *q*, the system aims to select a subset of relevant elements $\mathscr {C}_q \subseteq \mathscr {E}$ and generate an answer *y* that maximizes the expected reward:

2 $$\begin{aligned} (y^*, \mathscr {C}_q^*) = \arg \max _{y, \mathscr {C}_q} \mathbb {E}\big [R(\mathscr {C}_q, q, y)\big ]. \end{aligned}$$

The reward function $R(\cdot )$ quantifies answer quality, including accuracy, multimodal coverage, and interpretability.

This formulation integrates retrieval and reasoning under a unified objective. The hierarchical MARL module learns retrieval policies that adapt to both query semantics and modality characteristics, while the reasoning module provides feedback signals to refine agent behavior iteratively.

### Multimodal parsing module

The multimodal parsing module transforms a raw document *D* into a structured collection of layout-aware elements to facilitate downstream retrieval and reasoning. Formally, the document is parsed into

3 $$\begin{aligned} \mathscr {E} = \{ (e, \mathbb {T}(e), \text {meta}(e)) \mid e \in \text {Parse}(D) \}. \end{aligned}$$

where each element *e* is associated with a modality type $\mathbb {T}(e) \in \{\text {Text}, \text {Image}, \text {Table}\}$ and metadata

4 $$\begin{aligned} \text {meta}(e) = \big (p(e), \boldsymbol{b}(e), \ell (e), \phi (e)\big ). \end{aligned}$$

with *p*(*e*) denoting the page index, $\boldsymbol{b}(e)$ the normalized bounding-box coordinates, $\ell (e)$ the layout position, and $\phi (e)$ a functional role label such as title, caption, paragraph, or table header.

The parsing procedure proceeds in three steps. First, a region detection stage identifies textual, visual, and tabular regions at the page level, preserving their geometric relationships (https://github.com/allenai/olmocrhttps://github.com/allenai/olmocr). Second, optical character recognition is applied to text regions^39^ and table cells to extract token-level content, while table structure recognition recovers row and column organization^40^. Third, all detected elements are mapped to a common coordinate frame so that spatial relationships such as reading order, alignment, and adjacency can be consistently modeled across pages and modalities.

Each element is then encoded into a modality-aware embedding

5 $$\begin{aligned} \boldsymbol{v}(e) = f_{\text {enc}}\big (e, \mathbb {T}(e)\big ) + f_{\text {pos}}\big (p(e), \boldsymbol{b}(e), \ell (e)\big ) + f_{\text {tag}}\big (\phi (e)\big ). \end{aligned}$$

where $f_{\text {enc}}$ is a modality-specific encoder, $f_{\text {pos}}$ encodes page-level and geometric information, and $f_{\text {tag}}$ incorporates weak supervision from functional role labels. These embeddings jointly capture semantic, spatial, and structural cues, and serve as the unified representation for the candidate pool construction module and the hierarchical multi-agent Reinforcement Learning (RL) module.

### Candidate pool construction module

The candidate pool construction module takes as input the modality-aware embeddings $\{\boldsymbol{v}{(e)}\}_{e \in \mathscr {E}}$ produced by the parsing stage and builds modality-specific candidate sets for adaptive retrieval. Its goal is to map heterogeneous elements into a unified latent space to ensure cross-modal similarity can be measured in a consistent manner. The resulting modality-level candidate pools $\mathscr {C}_m$ and their union $\mathscr {C}$ serve as the search space for the hierarchical MARL module.

For each modality $m \in \{\text {Text}, \text {Image}, \text {Table}\}$, the embeddings are projected through a learnable transformation matrix $W_m$:

6 $$\begin{aligned} \boldsymbol{v}_m(e) = W_m \boldsymbol{v}(e), \quad e \in \mathscr {E}_m. \end{aligned}$$

where $\mathscr {E}_m$ denotes the set of elements with modality type *m*. This projection aligns modality-specific representations into a shared latent space while preserving modality-dependent structure.

Given a query *q*, a query encoder produces the query embedding $\boldsymbol{h}_q$. The semantic relevance between *q* and an element *e* of modality *m* is computed using cosine similarity:

7 $$\begin{aligned} s(e, q) = \frac{\boldsymbol{h}_q^\top \boldsymbol{v}_m(e)}{\Vert \boldsymbol{h}_q\Vert \,\Vert \boldsymbol{v}_m(e)\Vert }. \end{aligned}$$

Within each modality, the top-*K* elements with the highest similarity scores are selected to form a modality-specific candidate pool:

8 $$\begin{aligned} \mathscr {C}_m = \text {Top-}K\big (\mathscr {E}_m, s(\cdot , q)\big ). \end{aligned}$$

The global candidate set is obtained by taking the union over all modalities: $\mathscr {C} = \bigcup _{m \in \{T, I, B\}} \mathscr {C}_m$, where *B* represents the Table modality.

### Hierarchical multi-agent reinforcement learning module

The hierarchical multi-agent reinforcement learning module is responsible for adaptive retrieval over the multimodal candidate pools. Given the modality-specific candidate sets $\{\mathscr {C}_T, \mathscr {C}_I, \mathscr {C}_B\}$ constructed in the previous stage, this module learns (i) retrieval budgets per modality, (ii) context expansion depth, and (iii) termination timing. For each modality $m \in \{T, I, B\}$, the corresponding agent progressively constructs a selected subset $\mathscr {C}_m^*\subseteq \mathscr {C}_m$. At the end of retrieval, the final selections $\{\mathscr {C}_T^*, \mathscr {C}_I^*, \mathscr {C}_B^*\}$ together with the modality weights $(w_T, w_I, w_B)$ are passed to the collaborative reasoning module.

The module consists of one high-level coordinator agent and three modality-specific agents for text (*T*), images (*I*), and tables (*B*, for tabular Blocks). All agents are implemented as lightweight multi-layer perceptrons (MLPs) that operate on the embeddings of the candidate elements. The reinforcement learning signals for these agents are jointly derived from the retrieval accuracy and the quality of the final answers produced by the LLM.

We model the retrieval process as a hierarchical multi-agent Markov decision process

9 $$\begin{aligned} \mathscr {M} = (\mathscr {S}, \mathscr {A}_H, \{\mathscr {A}_m\}, P, R, \gamma ). \end{aligned}$$

where $\mathscr {S}$ is the state space, $\mathscr {A}_H$ is the action set of the coordinator, $\mathscr {A}_m$ is the action set of modality agent *m*, *P* is the state transition function, *R* is a hierarchical reward function, and $\gamma$ is a discount factor.

Coordinator agent At each time step *t*, the coordinator observes a compact summary of the current retrieval status:

10 $$\begin{aligned} \boldsymbol{s}_t^H = [\boldsymbol{h}_q,\ \boldsymbol{c}_t^T,\ \boldsymbol{c}_t^I,\ \boldsymbol{c}_t^B,\ \boldsymbol{g}_t]. \end{aligned}$$

where $\boldsymbol{h}_q$ is the query embedding, $\boldsymbol{c}_t^m$ is the aggregated representation (e.g., via mean-pooling) of the elements selected so far from modality *m*, and $\boldsymbol{g}_t$ encodes global metadata such as the current step *t* and task category.

Based on this state, the coordinator chooses one of the following high-level actions:

11 $$\begin{aligned} \mathscr {A}_H = \{UpdateWeights,\ IncreaseDepth,\ Terminate\}. \end{aligned}$$

The action ‘UpdateWeights’ re-calibrates the modality importance vector $(w_T, w_I, w_B)$, ‘IncreaseDepth’ allows additional retrieval steps, and ‘Terminate’ stops the retrieval process. The coordinator policy is parameterized by a neural network $\pi _H(a_t^H \mid \boldsymbol{s}_t^H; \theta _H)$ and is trained to dynamically allocate retrieval effort across modalities.

Modality agents For each modality *m*, a modality agent $A_m$ operates on its candidate pool $\mathscr {C}_m$. At time step *t*, its state vector is defined as

12 $$\begin{aligned} \boldsymbol{s}_t^m = [\boldsymbol{h}_q,\ \boldsymbol{v}_i^m,\ \boldsymbol{c}_t^m,\ \boldsymbol{u}_t^m]. \end{aligned}$$

where $\boldsymbol{v}_i^m$ is the embedding of a candidate element $e_i \in \mathscr {C}_m$, $\boldsymbol{c}_t^m$ is the aggregated representation of elements in $\mathscr {C}_m^*$, and $\boldsymbol{u}_t^m$ encodes local layout information. Conditioned on this state and the current modality weight $w_m$, the modality agent chooses one of the following actions:

13 $$\begin{aligned} \mathscr {A}_m = \{Select,\ Expand,\ Skip,\ Stop\}. \end{aligned}$$

The action ‘Select’ adds the current candidate element to $\mathscr {C}_m^*$, ‘Expand’ retrieves adjacent contextual elements, ‘Skip’ ignores the current candidate, and ‘Stop’ indicates that no further elements should be retrieved from modality *m*. Each modality agent is parameterized by $\theta _m$ and is trained to make fine-grained decisions.

Coordination and communication The agents interact in a bidirectional manner. The coordinator broadcasts the modality weights $(w_T, w_I, w_B)$ and the allowed retrieval depth to all modality agents. In return, each modality agent summarizes its current selection status via the updated state $\boldsymbol{c}_t^m$, which is folded into the global signal $\boldsymbol{g}_t$. This decentralized-execution scheme avoids complex message-passing mechanisms while still enabling cooperative retrieval.

Learning objective. All agents are trained jointly to maximize the expected cumulative reward

14 $$\begin{aligned} J(\Theta ) = \mathbb {E}\left[ \sum _{t=1}^{T} \gamma ^t \Big (R_t^{\text {retrieval}} + R_t^{\text {reasoning}}\Big )\right] . \end{aligned}$$

where $\Theta = \{\theta _H, \theta _T, \theta _I, \theta _B\}$ denotes the policy parameters, $R_t^{\text {retrieval}}$ measures retrieval quality (e.g., recall and precision), and $R_t^{\text {reasoning}}$ reflects the quality of the final answer produced by the collaborative reasoning module.

### Collaborative reasoning module

The collaborative reasoning module performs multimodal reasoning over the evidence selected by the MARL module. It takes as input the final modality-specific selections $\{\mathscr {C}_T^*, \mathscr {C}_I^*, \mathscr {C}_B^*\}$ and the learned modality weights $(w_T, w_I, w_B)$, and produces the final answer *y*.

After the coordinator executes the action Terminate, the selected elements are aggregated into a global evidence set

15 $$\begin{aligned} \mathscr {C}^*= \bigcup _{m \in \{T, I, B\}} \mathscr {C}_m^*. \end{aligned}$$

For each modality *m*, a summarization function $\Phi _m(\cdot )$ compresses the selected elements $\mathscr {C}_m^*$ into a compact representation $\boldsymbol{z}_m$ that is suitable for prompting, for example by concatenating key snippets or table rows. The LLM then receives the query together with the weighted multimodal summaries:

16 $$\begin{aligned} \text {Input}_{\text {LLM}} = \big \{\, q,\ (w_T, \boldsymbol{z}_T),\ (w_I, \boldsymbol{z}_I),\ (w_B, \boldsymbol{z}_B) \big \}. \end{aligned}$$

In our framework, the main reasoning component is powered by GPT-4o, while retrieval decisions are parameterized by policy networks.

To link reasoning quality back to retrieval, the module computes a scalar reasoning score $S(q, y, \mathscr {C}^*) \in \mathbb {R}.$ which quantifies how well the generated answer satisfies the task objective. During training, this score is used to construct the reasoning reward $R_t^{\text {reasoning}}$ to optimize the retrieval policy via policy gradients.

The collaborative reasoning module also supports an iterative self-correction mechanism. Starting from the initial evidence set $\mathscr {C}^{(0)} = \mathscr {C}^*$, the LLM produces an intermediate answer $y^{(k)}$ and obtains a reasoning score $S^{(k)}$. If the score is above a threshold $\eta$, the current answer is accepted. Otherwise, the system triggers an additional retrieval step:

17 $$\begin{aligned} \mathscr {C}^{(k+1)} = {\left\{ \begin{array}{ll} \mathscr {C}^{(k)}, & S^{(k)} \ge \eta . \\ \mathscr {C}^{(k)} \cup \Delta \mathscr {C}^{(k)}, & S^{(k)} < \eta . \end{array}\right. } \end{aligned}$$

where $\Delta \mathscr {C}^{(k)}$ denotes supplementary content proposed by the modality agents.

Through this iterative retrieve–reason–reflect procedure, the module closes the loop between retrieval and generation, leading to more faithful and well-grounded multimodal document understanding.

**Algorithm 1:**
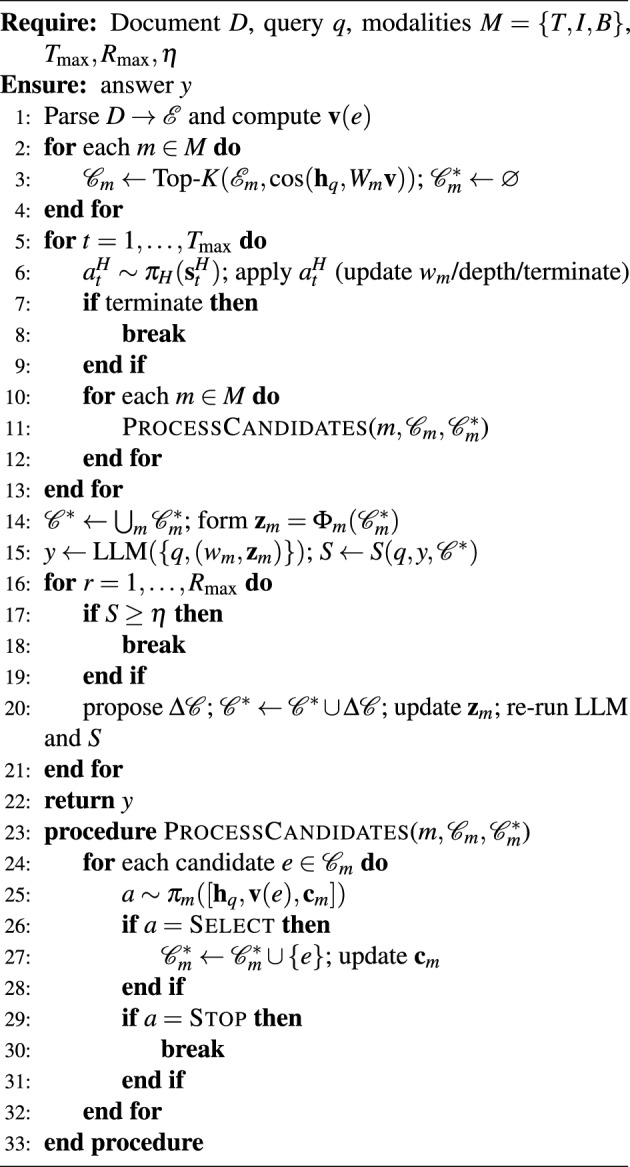
MARL-RAGDoc

## Experiment

### Datasets

To evaluate the effectiveness of MARL-RAGDoc in long-context document understanding and RAG tasks, we employ three heterogeneous multi-modal datasets. These include two widely recognized public benchmarks and one domain-specific corpus curated from real-world documents in the power industry. This selection ensures a comprehensive evaluation across generic long-context tasks, industrial scanned document analysis, and specialized technical reasoning. Dataset statistics are summarized in Table 1.

**MMLongBench-Doc**^41^ is a multi-modal benchmark designed for long-context document understanding. Notably, approximately 22.8% of the questions are designed to be unanswerable, serving as a rigorous testbed for evaluating the model’s robustness against hallucination.

**MP-DocVQA**^42^ is a visual question answering dataset tailored for multi-page industrial scanned documents. It extends traditional single-page document tasks by requiring models to navigate complex spatial layouts and cross-page context.

**PowerMM-Doc**. We constructed PowerMM-Doc (Power Multi-Modal Document Dataset), a multi-modal long-context dataset that encapsulates the complex retrieval-and-reasoning needs of real-world power sector scenarios. The dataset includes diverse technical documents such as design specifications, operational guidelines, equipment manuals, and technical reports in heterogeneous formats (e.g., PDF, Excel, PPT, and scanned images). To ensure high-fidelity structural representation, all documents underwent a rigorous preprocessing pipeline, including page segmentation, OCR, layout analysis, and the extraction of fine-grained multi-modal elements such as text paragraphs, equipment images, tables, and procedural flowcharts.

Based on this, question-answer pairs were developed through a human-in-the-loop approach: they were initially synthesized semi-automatically and then meticulously verified by domain experts to ensure technical accuracy. These pairs cover sophisticated tasks including cross-page retrieval, cross-modal reasoning, and multi-step process logic understanding. Additionally, unanswerable questions are incorporated to evaluate grounding performance. PowerMM-Doc is comparable to SOTA benchmarks in scale and complexity while introducing unique challenges inherent to specialized technical domains.

**Table 1:** Summary of three multi-modal document datasets.

	MMLongBench-Doc	MP-DocVQA	PowerMM-Doc
# Documents	130	5929	1200
Avg. Length (pages)	49.4	47	64.8
# Questions	1062	46346	13672
Modalities	Text, image, chart, table, layout structure	Text, image, chart, table, layout structure	Text, image, table, chart, flow diagram, Layout Structure
Formats	PDF	PDF, Excel, PPT	PDF, Excel, PPT, Scanned Images

“#” denotes “number of”.

### Baselines

To comprehensively benchmark MARL-RAGDoc against state-of-the-art (SOTA) solutions on long-context document understanding and RAG tasks, we categorize and adopt several representative baselines. These models are grouped into: (i) general-purpose multimodal LLMs (MLLMs), (ii) layout-aware or specialized parsing-based RAG systems, and (iii) agentic multimodal RAG frameworks. These include MLLMs such as Qwen2-VL and ChatGPT-4o, as well as specialized systems such as LayoutLMv3, TabRAG, M3DocRAG, HM-RAG, and VisDoM.

**Qwen2-VL**^43^ is a SOTA open-source vision-language model optimized for document-level tasks, capable of processing both OCR-based textual features and raw visual content. We utilize it as a representative for direct multimodal inference.

**ChatGPT-4o**^44^ is a proprietary large model with powerful cross-modal understanding and reasoning capabilities. It serves as a strong general-purpose baseline to establish a performance benchmark for zero-shot reasoning.

**LayoutLMv3**^1^ is a multi-modal pre-trained model that integrates text and images through unified objectives (e.g., MLM, MIM, and WPA). It is included as a layout-aware baseline to evaluate the modeling of geometric and structural relationships.

**TabRAG**^45^ is a specialized RAG pipeline optimized for table-heavy documents, leveraging structured language representations instead of high-cost fine-tuning. It is employed to assess performance in table-dominant scenarios.

**M3DocRAG**^46^ is a multi-modal RAG approach designed for multi-page scenarios, capable of retrieving and reasoning over heterogeneous evidence. It represents a robust baseline for cross-page information aggregation.

**HM-RAG**^47^ is a hierarchical multi-agent framework featuring a three-tiered architecture for query decomposition, parallel multi-source retrieval, and decision-based refinement. It serves as an agentic baseline to evaluate coordinated reasoning across heterogeneous data.

**VisDoM**^5^ is a dual-stream RAG system that parallelizes textual and visual retrieval pipelines, resolving cross-modal discrepancies through a consistency-constrained fusion module. It benchmarks modality-specific evidence integration in visually rich documents.

### Implementation details

Our MARL-RAGDoc model is implemented in PyTorch. The hierarchical multi-agent reinforcement learning module uses the Adam optimizer with a learning rate of $1 \times 10^{-4}$ and a weight decay of $1 \times 10^{-5}$. The neural policy networks for the coordinator and each modality-specific agent are two-layer feed-forward networks with 256 hidden units per layer. Modality-specific features are projected into a 128-dimensional latent space for retrieval. The initial Top-K values for candidate pool construction are set to 10 for text, 8 for tables, and 6 for images. The discount factor $\gamma$ for reinforcement learning is set to 0.95.

The collaborative reasoning module leverages a LLM to generate final answers based on the weighted multimodal evidence summaries. A reflection threshold $\tau$ is used to determine whether additional retrieval steps are needed, forming the retrieve–reason–reflect loop. The reward signals for the agents combine retrieval quality (relevance and multimodal coverage) with reasoning performance, which is evaluated based on the LLM-generated answer quality. All experiments are conducted on two NVIDIA A5000 GPUs.

**Table 2:** Experimental results of MARL-RAGDoc against baseline methods. Bold values indicate the overall best performance across all methods, while underlined values represent the best-performing baselines among all competitors.

Methods	MMLongBench-Doc			MP-DocVQA			PowerMM-Doc		
	ACC	EM	MRR	ACC	EM	MRR	ACC	EM	MRR
Qwen2-VL^43^	6.70	5.80	6.20	6.10	5.40	5.70	6.30	5.20	5.70
GPT-4o^44^	40.29	42.67	41.48	34.40	30.20	32.30	35.05	32.09	33.57
LayoutLMv3^1^	41.49	43.61	42.55	43.27	44.46	43.86	39.65	41.30	40.47
TabRAG^45^	44.71	43.85	44.28	43.26	**45.17**	44.22	40.73	42.00	41.36
M3DocRAG^46^	32.73	34.66	33.70	22.50	23.58	23.04	37.65	36.28	36.97
HM-RAG^47^	47.85	45.10	45.30	44.92	42.85	44.60	44.20	43.50	44.15
VisDoM^5^	46.88	44.51	45.12	45.35	43.10	44.85	43.53	41.87	42.96
**MARL-RAGDoc**	**50.36**	**47.52**	**46.94**	**46.59**	43.74	**45.16**	**48.67**	**46.14**	**47.41**

### Metric

To evaluate model performance in multimodal document understanding and question-answering tasks, we adopt three widely used metrics: Accuracy (ACC), Exact Match (EM), and Mean Reciprocal Rank at Top-10 (MRR@10).

ACC reflects the overall proportion of correctly answered queries, providing a general measure of predictive performance.

EM assesses whether the predicted answer matches the ground truth exactly, making it a stricter indicator that does not permit partial correctness:

18 $$\begin{aligned} \text {EM} = \frac{M}{N}, \end{aligned}$$

where *M* denotes the number of exact matches and *N* is the total number of samples.

MRR quantifies how early the first correct answer appears in the ranked predictions. MRR@10 restricts the evaluation to the top-10 outputs:

19 $$\begin{aligned} \text {MRR@10} = \frac{1}{Q} \sum _{i=1}^{Q} \frac{1}{\text {rank}_i}, \end{aligned}$$

where *Q* is the total number of queries and $\text {rank}_i$ is the position of the first correct answer for query *i* within the top-10 predictions.

### Main results

Experimental results across the three multimodal document benchmarks, as shown in Table 2, demonstrate that MARL-RAGDoc achieves superior overall performance, outperforming baselines across the vast majority of key metrics. Compared with the best-performing baseline on each dataset, MARL-RAGDoc yields absolute improvements of approximately 5–9% in ACC, 4–8% in EM, and 4–7% in MRR@10, reflecting substantial gains in both retrieval relevance and downstream reasoning accuracy. These improvements are particularly pronounced in long-document and cross-modal scenarios, where conventional systems often fail to capture dispersed or structurally complex evidence.

General-purpose MLLMs such as Qwen2-VL and GPT-4o exhibit notable degradation when applied to multi-page industrial documents. Their constrained context windows and lack of explicit modeling for layout structures or tabular semantics frequently result in truncated evidence or spurious context. As a result, their performance trails MARL-RAGDoc by over 10–20% across several metrics, underscoring the limitations of purely generative or monolithic architectures in evidence-intensive tasks.

Document-oriented RAG baselines exhibit varying degrees of effectiveness depending on their architectural sophistication. Traditional approaches, specifically LayoutLMv3, TabRAG, and M3DocRAG, provide stable gains through structure-aware encoding but often rely on static fusion strategies or fixed-length constraints. Consequently, they typically fall 5–10% below MARL-RAGDoc in ACC. While TabRAG shows competitiveness in specific table-centric metrics (e.g., EM on MP-DocVQA), it lacks the cross-modal adaptability required for more complex tasks. More recent advanced frameworks like HM-RAG and VisDoM demonstrate stronger competitiveness by adopting hierarchical agentic or dual-stream architectures, effectively narrowing the performance gap to 2–5%. However, even these state-of-the-art methods rely primarily on rule-based decomposition or late-stage consistency checks. These mechanisms tend to introduce redundancy and lack the flexibility to dynamically optimize retrieval budgets based on real-time reasoning feedback, which remains a core advantage of our learning-driven MARL approach.

By contrast, MARL-RAGDoc’s hierarchical multi-agent design enables adaptive allocation of retrieval depth and modality importance. The coordinator adjusts high-level retrieval strategies based on semantic cues, while modality-specific agents perform fine-grained evidence refinement through selection, expansion, and filtering actions. This coordinated mechanism effectively mitigates redundancy while enhancing cross-modal complementarity, thereby yielding higher-quality evidence for downstream reasoning. The resulting performance gains surpass advanced agentic baselines such as HM-RAG and VisDoM, which highlights the efficacy of closing the loop between retrieval agents and reasoning feedback within complex industrial document environments.

### Ablation study

#### Influence of the coordinator agent

**Table 3:** Accuracy comparison of different coordinator configurations.

Datasets	No coordinator	Simple coordinator	MARL-RAGDoc
MMLongBench-Doc	34.77	41.19	**50.36**
MP-DocVQA	32.19	38.94	**46.59**
PowerMM-Doc	35.27	40.34	**48.67**

To evaluate the contribution of the high-level Coordinator agent within the hierarchical retrieval strategy, we compare three configurations: No Coordinator (where modality agents operate independently), Simple Coordinator (which provides fixed or rule-based modality weights and coarse retrieval control), and MARL-RAGDoc (the full hierarchical Coordinator). As shown in Table 3, consistent performance gaps emerge across all three datasets.

Performance degrades significantly when the Coordinator is removed. Without global guidance, each modality agent relies solely on local similarity signals to make retrieval decisions, which often leads to unbalanced retrieval depths, over-reliance on a single modality, or the omission of key cross-modal evidence. Consequently, the reasoning stage suffers from incomplete or insufficient complementary evidence.

Introducing a simple Coordinator yields moderate improvements, indicating that lightweight global weighting can partially alleviate retrieval bias. However, as this baseline Coordinator cannot adaptively respond to query semantics, retrieval progress, or dynamic inter-modal dependencies, its coordination capacity remains limited and provides only coarse-grained global balance.

In contrast, the full hierarchical Coordinator achieves the optimal performance across all datasets. By monitoring retrieval progress, cross-modal evidence coverage, and the remaining retrieval budget, it dynamically adjusts modality weights and retrieval depth allocations. This enables our method to significantly enhance the completeness and coherence of cross-modal evidence, thereby aligning retrieval behavior more effectively with the requirements of downstream reasoning.

Overall, these findings demonstrate that the high-level Coordinator not only optimizes resource allocation during retrieval but also substantially bolsters the system’s capacity for complex cross-modal reasoning.

#### Influence of modality agents

**Table 4:** Accuracy comparison of modality agent integration in multimodal retrieval systems.

Datasets	w/o modality agents	MARL-RAGDoc
MMLongBench-Doc	38.55	**50.36**
MP-DocVQA	37.21	**46.59**
PowerMM-Doc	35.02	**48.67**

To assess the functional role of modality agents in the multimodal retrieval pipeline, we construct a simplified ablation variant by deactivating all three modality agents. In this setting, the system relies solely on a static Top-K retrieval strategy and directly invokes a large language model for answer generation. Compared with the full system, this simplified version lacks dynamic selection and context-expansion capabilities at the modality level, reducing the retrieval stage to a rigid and coarse-grained evidence construction process.

Experimental observations reported in Table 4 indicate a clear degradation in downstream reasoning performance without the modality agents. This is primarily because static Top-K retrieval is insufficient for capturing local structural dependencies in long documents and cannot establish stable semantic complementarity across modalities. As a result, irrelevant or redundant content is frequently retained, while critical cross-region evidence is inadequately captured. In contrast, modality agents leverage reinforcement learning to make fine-grained, state-dependent decisions–such as selecting, expanding, or skipping candidates–thereby constructing compact, relevant, and cross-modally coherent evidence sets.

In summary, the modality agent module equips each modality with learnable, fine-grained retrieval control, enabling the system to assemble high-quality and semantically aligned multimodal evidence. This capability is a pivotal component underpinning the robust reasoning performance of our approach.

### Impact of top-K retrieval size

**Fig. 3:**
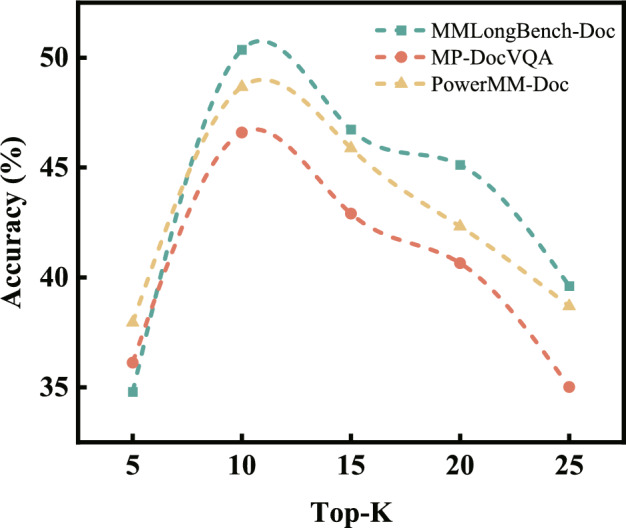
Impact of retrieval depth on model performance across datasets.

To examine how the size of the retrieval candidate pool affects the system’s reasoning performance, we conducted a sensitivity study by varying the Top-K parameter. In this setting, the retrieval pipeline remains fixed, without any adaptive expansion or modality-specific selection mechanisms. Only the candidate pool size is changed to isolate its influence on performance. We evaluate $K \in \{5, 10, 15, 20, 25\}$ on three datasets and report the corresponding accuracy of the downstream reasoning model in Fig. 3.

Across all datasets, the results exhibit a consistent “increase–peak–decline” pattern. When K grows from 5 to 10, performance improves substantially, indicating that small candidate pools often suffer from insufficient recall, failing to include key evidence. Expanding K moderately increases the probability of retrieving relevant segments, thereby enhancing answer completeness and stability. However, when K exceeds 10, accuracy gradually decreases. Larger candidate pools tend to introduce additional irrelevant or weakly correlated content, which makes the evidence set noisier and complicates reasoning.

Overall, K=10 yields the best performance across datasets, suggesting that a moderately sized candidate pool provides a balanced trade-off between recall and noise control. Very small K values lead to incomplete evidence coverage, whereas excessively large K values accumulate noise and diminish retrieval effectiveness. These findings confirm that retrieval depth is not monotonically beneficial; it must be tuned to the characteristics of the task.

In summary, the choice of Top-K has a notable impact on retrieval-driven document reasoning. A compact but sufficiently informative candidate set is crucial for maintaining evidence relevance and supporting accurate downstream reasoning. Based on these observations, our default configuration adopts K=10, as it consistently offers a favorable balance between retrieval cost and accuracy.

### Impact of the discount factor

**Fig. 4:**
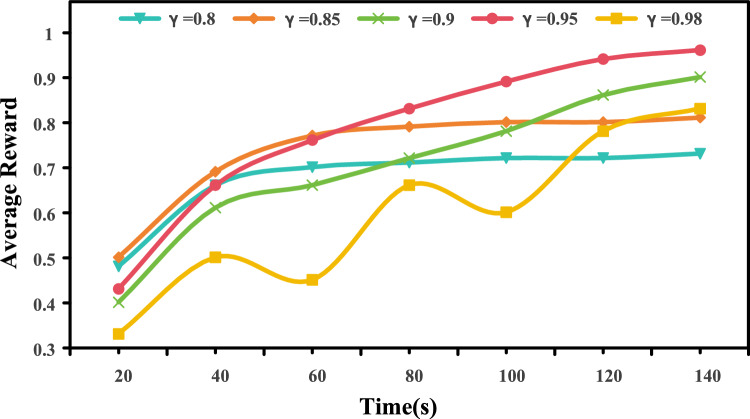
Training convergence under different discount factors $\gamma$. Average normalized reward is plotted against training time.

We study the effect of the discount factor $\gamma$ on training dynamics by varying $\gamma \in \{0.8, 0.85, 0.9, 0.95, 0.98\}$ while keeping all other hyperparameters fixed. Figure 4 plots the average normalized reward over training time (higher is better).

All settings improve rapidly at the early stage, indicating stable policy learning. However, convergence speed and final reward differ across $\gamma$. $\gamma =0.95$ converges quickly and achieves the highest final reward, suggesting a good balance between short- and long-term returns. Smaller values (e.g., $\gamma =0.8, 0.85$) rise faster initially but plateau earlier, indicating weaker long-horizon optimization. In contrast, a larger value ($\gamma =0.98$) introduces noticeable oscillations, likely due to higher variance in long-horizon credit assignment, and thus requires longer training to stabilize. Overall, $\gamma =0.95$ provides the best trade-off between convergence speed, stability, and final performance.

**Table 5:**
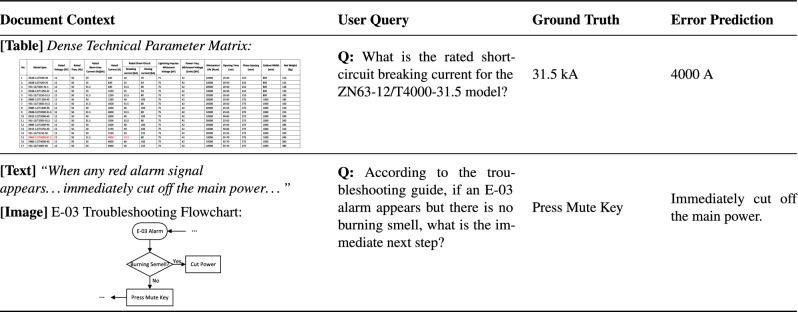
Failure case analysis of our MARL-RAGDoc framework when processing the PowerMM-Doc dataset.

### Time complexity

**Fig. 5:**
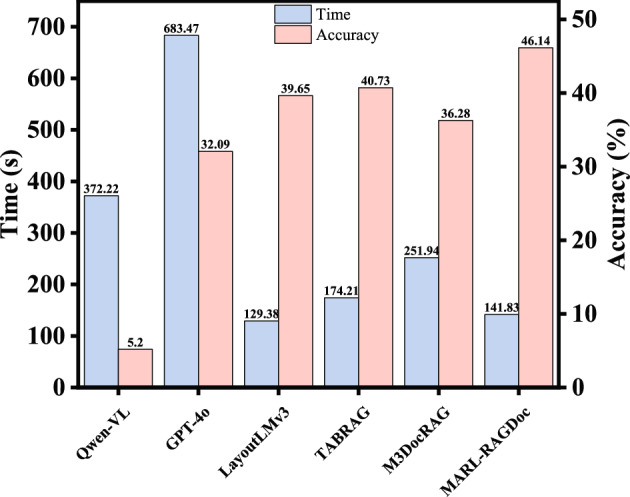
Average retrieval time (s) and ACC (%) of different methods on the PowerMM-Doc dataset.

Under a unified experimental setup, we report both the average retrieval time and the corresponding accuracy for each method to evaluate their efficiency and overall capability, as shown in Fig. 5. Traditional document understanding models (e.g., LayoutLMv3) employ relatively fixed processing pipelines with shorter feature-encoding and retrieval paths, resulting in low inference latency with minimal variance. However, their accuracy remains constrained by the limitations of unimodal modeling, yielding only moderate performance.

TabRAG, which incorporates structured layout alignment, introduces additional layout parsing and candidate re-ranking operations during retrieval. These steps moderately increase inference time while offering corresponding gains in accuracy. M3DocRAG further extends the retrieval workflow through cross-modal graph construction and multi-stage retrieval. The associated graph-building and relation-updating processes impose substantial computational overhead, leading to noticeably higher inference costs, while its accuracy can be affected by the stability of the multi-stage reasoning procedure.

Large multimodal models (such as GPT-4o and Qwen2-VL) require joint processing of textual and visual inputs within a unified architecture. This design results in the highest latency among all evaluated systems, and the improvement in retrieval accuracy does not scale proportionally with the increased computational demand.

In contrast, the proposed MARL-RAGDoc reduces inference overhead by avoiding redundant interaction paths and employing a lightweight coordination mechanism, while maintaining effective cross-modal reasoning. Experimental results show that the method achieves markedly higher accuracy than traditional document models, with an inference time close to theirs. Overall, MARL-RAGDoc achieves a more favorable balance between computational efficiency and retrieval performance.

### Error analysis

To illustrate the limitations of our method, we present two representative failure cases from the PowerMM-Doc dataset in Table 5.

The first case is a fine-grained lookup in a dense technical parameter table. The query asks for the rated short-circuit breaking current of the ZN63-12/T4000-31.5 model. Although the system retrieves the correct table row, it outputs 4000 A instead of the ground truth 31.5 kA. This error is mainly caused by fine-grained cell selection: despite identifying the target row, the Table Agent extracts a value from the adjacent “rated current” column rather than the intended “short-circuit breaking current” column, likely due to tight column spacing and weak gridlines in the scanned table.

The second case reflects a reasoning error under conflicting cross-modal evidence. The document contains a general instruction in the text section (cut off the main power upon any alarm) and a more specific troubleshooting flowchart for the E-03 alarm (press the Mute key). When asked for the immediate next step for E-03 without a burning smell, the model follows the general textual instruction instead of the flowchart. This suggests that the reasoning module does not consistently enforce a specificity hierarchy, where procedure-specific visual evidence (e.g., flowcharts) should override generic textual guidelines when they conflict.

## Discussion and limitations

Our results show that MARL-RAGDoc improves multimodal document QA through adaptive modality–depth control, modality-specific evidence selection/expansion, and a retrieve–reason–reflect loop that refines evidence when initial reasoning is insufficient. Although we mainly evaluate on power-industry documents, the framework is largely domain-agnostic because it operates on generic document elements (text, images, tables) with query-conditioned coordination. We expect it to transfer to other industrial domains (e.g., manufacturing, healthcare, aerospace), potentially benefiting from domain adaptation such as fine-tuning modality encoders.

Limitations remain. (1) Fine-grained visual queries (e.g., small text in diagrams) may fail when the image agent selects overly coarse regions (about 8% of image-heavy queries), suggesting hierarchical region decomposition or zoom-in attention. (2) Very long documents (>100 pages) can trigger premature stopping under a fixed budget (about 5%), calling for length-/density-aware budget scheduling. (3) Low-quality OCR degrades text retrieval (about 3%); incorporating OCR confidence and stronger preprocessing may improve robustness. Finally, our training currently uses supervised rewards from ground-truth answers; extending MARL-RAGDoc to weakly supervised or self-supervised rewards is an important direction for low-resource settings.

## Conclusions

In this paper, we presented MARL-RAGDoc, a hierarchical multi-agent reinforcement learning framework designed to address the limitations of static retrieval strategies in multimodal document understanding. The framework allows a high-level coordinator to dynamically adjust modality importance and retrieval depth, while specialized modality agents execute fine-grained selection and expansion actions. Through reward-guided optimization, retrieval behavior becomes aligned with downstream reasoning quality. Experiments on several multimodal document benchmarks demonstrate that MARL-RAGDoc consistently outperforms SOTA baselines while maintaining competitive inference efficiency. Future work will explore support for additional modalities, self-supervised reward mechanisms for low-resource scenarios, and enhanced communication strategies among agents.

## Data Availability

The data that support the findings of this study are available from State Grid Corporation of China but restrictions apply to the availability of these data, which were used under license for the current study, and so are not publicly available. Data are however available from the authors upon reasonable request and with permission of State Grid Corporation of China.
